# RNA-Seq Analysis of the Liver Transcriptome Reveals the Networks Regulating Treatment of Sitagliptin Phosphate plus Fuzhujiangtang Granule in the Zucker Diabetic Fatty Rats

**DOI:** 10.1155/2020/8463858

**Published:** 2020-04-13

**Authors:** Xuan Guo, Wen Sun, Guangyuan Xu, Dan Hou, Zhuo Zhang, Lili Wu, Tonghua Liu

**Affiliations:** ^1^Dongfang Hospital of Beijing University of Chinese Medicine, Beijing 100078, China; ^2^Key Laboratory of Health Cultivation of the Ministry of Education, Beijing University of Chinese Medicine, Beijing 100029, China; ^3^Beijing Key Laboratory of Health Cultivation, Beijing University of Chinese Medicine, Beijing 100029, China; ^4^Department of Traditional Chinese Medicine, Fu Xing Hospital of Capital Medical University, Beijing 100045, China

## Abstract

Diabetes is one of the most serious chronic diseases. Numerous drugs including oral agents and traditional Chinese medicines, such as sitagliptin phosphate (SP) and Fuzhujiangtang granules (FJG), have been discovered to treat diabetes and used in combination in clinical practice. However, the exact effect and underlying mechanism of using combined medicine is not clear. In this study, we compared the antidiabetic effect of SP, FJG, and SP plus FJG (SP-FJG) using forty 8-week-old Zucker diabetic fatty (ZDF) rats and 10 age-matched Zucker lean rats as the normal control group. ZDF rats were treated with different therapies, respectively, for 6 weeks. The study showed that the fast blood glucose, random blood glucose (RBG), oral glucose tolerance test (OGTT), insulin tolerance test (ITT), homeostasis model of assessment-insulin resistance index, triglyceride (TC), superoxide dismutase, and malondialdehyde of each treatment group were improved when compared with the diabetes mellitus (DM) control group. Using SP-FJG in combination had better improvements in OGTT, fast serum insulin levels, TNF-α, and IL-6 compared with using SP individually. Besides, the increased LDL and TC caused by using SP was attenuated by using FJG in combination. Meanwhile, compared with the DM group, 1781 differentially expressed genes (DEGs) (including 1248 mRNA, 211 ncRNA, 202 cirRNA, and 120 miRNA) were enriched in 58 pathways. Through analysis of ceRNA networks, we found that *rno-miR-326-3p*, *rno-miR-423-5p*, *rno-miR-15b-5p*, *rno-let-7c-5p*, and *rno-let-7b-5p* were related to pharmacodynamics in different groups. By analyzing the protein-protein interaction (PPI) and coexpression networks of the transcriptomes of different groups, it is inferred that *Lrrk2* and *Irak3* may be pharmacodynamic genes for type 2 diabetes mellitus (T2DM). Our research compared the treatment of SP, FJG, and SP-FJG and acquainted the PPI network, coexpression network, mutations, and pharmacodynamics genes, which reveals the new mechanisms of pathogenesis of T2DM.

## 1. Introduction

Worldwide, the prevalence of chronic noncommunicable diseases is growing at a phenomenal rate [1]. A growing number of people get diabetes as a result of population growth, aging, urbanization, and increasing prevalence of obesity and lack of exercises [2]. Throughout the world, the number of people with diabetes is estimated to increase from 171 million in 2000 to 366 million by 2030. Furthermore, this increase will be most evidenced in developing countries, where the number of people with diabetes is expected to increase from 84 million to 228 million. Similarly, diabetes is becoming more and more serious in China, and the study of the prevalence of diabetes showed that the total proportion of diabetes patients was 9.7% (10.6 percent for men and 8.8 percent for women, 50.2 million men and 42.2 million women) [3].

Now, some oral agents such as sulfonylureas, metformin, sitagliptin phosphate (SP), and injection of insulin are extensively used in the treatment of type 2 diabetes mellitus (T2DM). In the United States, five classes of oral agents (sulfonylureas, metformin, acarbose, troglitazone, and repaglinide) with different mechanism of action are currently available to improve glycemic control in patients with T2DM [4]. Besides, four classes of new agents are available on glycemic in T2DM, including the glucagon-like peptide-1 (GLP-1) analogue exenatide, dipeptidyl peptidase-4 (DPP-4) inhibitors sitagliptin and vildagliptin, and the long-acting insulin analogues, glargine and detemir [5]. In addition, many traditional Chinese medicines are also been reported to be used in the treatment of T2DM. Buddleia flower (Mi-Meng-Hua in Chinese) [6], Szechuan Lovage Rhizome (Chuan-Xiong in Chinese) [7], *Rehmannia* (Di-Huang in Chinese) [8], and *Coptis* (Huang-Lian in Chinese) [9] were studied that confirmed these herbs can be used alone or in conjunction with other herbs to enhance the therapeutic effects. Furthermore, the GC-TOF/MS analysis and sequencing technology were used to study the therapeutic action of MDG-1, a water-soluble *β*-d-fructan polysaccharide from *O. japonicus* [10], and Tangnaikang [11] in T2DM.

Numerous research studies of noninsulin-dependent diabetes mellitus have been described over the years [12, 13]. In the past several years, the Zucker diabetic fatty (ZDF) rat provides a model for human T2DM. The ZDF rat carries a spontaneous mutation in the leptin receptor (*fa* gene) which was originally derived from the Zucker fatty rat [14].

Recently, a certain amount of research studies were performed to explore the expression profiles of lncRNAs (noncoding RNA range from 200 nt to 100 kb) in different diseases besides T2DM which enriched the raw data in studying its primary functions [13–15]. The regulatory effect of lncRNAs is realized by a large complex network that involves mRNAs, miRNAs, and proteins rather than solitary [16]. Circular RNAs (circRNAs) is another group of noncoding RNAs that are widely distributed in animal cells. It is similar to lncRNAs, and studies showed the expression of circRNAs in different cell types with different parameters indicating its possible regulatory function [16, 17]. To date, there are few studies focusing on the role of lncRNA and circRNAs in treating T2DM with combination of Western medicine and traditional herbal medicine.

In the present study, we performed microarray analysis on the expression profiles of lncRNAs, mRNAs, circRNAs, and miRNAs using ZDF rats, a model of T2DM which was given different treatment. Gene ontology (GO) and Kyoto Encyclopedia of Genes and Genomes (KEGG) pathway analysis were done based on the function of mRNAs that their expression levels changed with lncRNAs's expression in a positive or negative correlation. The coexpression network was constructed according to the sequencing results and bioinformatics predictions, which was to reflect the potential targeting relationship.

## 2. Materials and Methods

### 2.1. Animals

Eight-week-old ZDF rats were feeding rodent chow (Purina #5008, Harlan Teklad, Indianapolis, IN) for 4 weeks. 40 rats with blood glucose >11.1 mM were identified as successful diabetes mellitus models. A normal control group (NC) of age-matched Zucker lean control rats was also included in the study and fed a standard laboratory chow diet throughout the study. All rats were housed under controlled conditions (12 : 12-h light-dark cycle, 24°C, and 50% relative humidity) with free access to water and food according to a protocol approved by the Beijing University of Medicine Animal Care Committee.

### 2.2. Herbs and Reagents

Fuzhujiangtang granule (FJG) was made of *Momordica charantia* (Ku-Gua in Chinese), *Polygonatum odoratum* (Yu-Zhu in Chinese), *Morus alba L.*(Sang-Ye in Chinese), *Panax notoginseng* (San-Qi in Chinese), and *Cinnamomum cassia Presl* (Rou-Gui in Chinese) with a ratio 1 : 1.33 : 1.07 : 0.27 : 0.07. The *Momordica charantia* granule was produced by Tian Yi Bio-pharmaceutical Co. Ltd. Other granules were produced by Sun Ten pharmaceutical Co. Ltd. (purchased from the Dongfang Hospital of Beijing University of Chinese Medicine). The granules were mixed into deionized water and stored at 4°C before use. SP (MSD, USA) tablets were dissolved in the deionized water and stored at 4°C before use.

### 2.3. Experimental Design

40 successful diabetes mellitus model rats were divided into the following groups and treated as indicated: diabetes mellitus group (DM) group (deionized water), SP group (SP [9 mg/kg·d^−1^]), FJG group (FJG [0.64 g/kg.d^−1^]), and SP-FJG group (SP [9 mg/kg·d^−1^] and FJG [0.64 g/kg·d^−1^]). Besides, the NC group was intragastrically administered with the same volume of deionized water. All treatments were given via oral gavage once a day, while the SP-FJG group was given FJG at 9am and SP at 9pm a day. The drugs were orally administered for 6 weeks in different groups.

### 2.4. Measurement of Body Weight, Blood Glucose, Oral Glucose Tolerance Test (OGTT), and Insulin Tolerance Test (ITT)

The activity, diet, and body posture of rats were recorded. Weekly body weight monitoring, FBG (fasting blood glucose), and RBG (random blood glucose) were measured at 7am to 9 am.

OGTT was measured at the sixth week of administration, and the area under the curve (AUC) was calculated. The rats were fasted for 12 h, and 50% glucose solution was used for intragastric administration according to 2 g/kg criteria. Blood glucose value was measured with the intragastric administration before (0 min) and 30, 60, and 120 min after. The formula for AUG calculation for blood glucose (BG) levels observed during the OGTT is as follows: AUC = 0.5 × (BG 0 min + BG 30 min)/2 + 0.5×(BG 30 min + BG 60 min)/2 + 1 × (BG 60 min + BG 120 min)/2.

Rats were given the ITT test on the last day of 6 weeks of administration, and 2 U/kg insulin (Humulin R, Novo Nordisk, Denmark) was subcutaneously injected in rats, and hypoglycemic effect was observed at 0 min, 30 min, 60 min, and 120 min. Also AUC was calculated according to the above formula.

### 2.5. Blood Biochemical Index

The fast serum insulin levels (FINS, mIU/L) were measured with an enzyme-linked immune sorbent assay (ELISA) using a rat insulin ELISA kit (Alpco, USA). Tumor necrosis factor-*α* (TNF-*α*, pg/ml) and interleukin-6 (IL-6 pg/ml) were determined using ELISA kits for rat, respectively (SINO-UK Institute of Bio-Tech, China). High-density lipoprotein (HDL, mmol/L), low-density lipoprotein (LDL, mmol/L), total cholesterol (TC, mmol/L), and triglyceride (TG, mmol/L) were measured by specific kits (Zhong Sheng Bei Kong, China), and superoxide dismutase (SOD, U/ml) and malondialdehyde (MDA, nmol/ml) were analyzed by kits (Nanjing Jiancheng, China). All assays were performed according to the manufacturers' recommendations. Insulin sensitivity was assessed using the homeostasis model of assessment-insulin resistance index (HOMA-IR), which was calculated using the following equation: HOMA-IR = FBG (mmol/L) × FINS (mIU/L)/22.5.

### 2.6. Sample Preparation

Total RNA was extracted from the liver by the Trizol reagent (Invitrogen) separately. The RNA quality was checked by Bioanalyzer 2200 (Aligent) and kept at −80°C. The RNA with RNA integrity number (RIN) > 8.0 is right for rRNA depletion. The RNA with RIN >8.0 is right for miRNA purification. The miRNA was purified by miRNeasy Mini Kit (Qiagen), and the purification result was validated by gel electrophoresis.

### 2.7. cDNA Library Construction

The cDNA libraries were constructed for each pooled RNA sample using the VAHTSTM Total RNA-seq according to the manufacturer's instructions. The tagged cDNA libraries were pooled in equal ratio and used for 150 bp paired-end sequencing in a single lane of the Illumina HiSeqTM 2500 with 51 plus 7 cycles by NovelBio Corp. Laboratory, Shanghai.

### 2.8. miRNA Library Construction and RNA Sequencing

The complementary DNA (cDNA) libraries for single-end sequencing were prepared using Ion Total RNA-Seq Kit v2.0 (Life Technologies) according to the manufacturer's instructions. After enrichment, the mixed template-positive Ion PITM Ion SphereTM Particles of samples was loaded on to 1 P1v2 Proton Chip (Life Technologies) and sequenced on Proton Sequencers according to Ion PI Sequencing 200 Kit v2.0 (Life Technologies) by NovelBio Corp. Laboratory, Shanghai.

### 2.9. Prediction of circRNA

We use the special splicing form of circRNA in the expression process to forecast the sequencing reads and to find such a read: covering two exons and the direction is opposite to the linear RNA, that is, the possible circRNA in the sequencing sample is obtained.

### 2.10. Differential Expression Analysis

We analyzed the differentially expressed circRNA, mRNA, and miRNA based on the data obtained by sequencing. The different mRNA, circRNA, and miRNA were selected by an international universal differential screening algorithm EB. The difference screening criteria were fold change >1.5, fold change <0.667, and FDR < 0.05. Differential mRNA and differential ncRNA were identified according to the gene type annotation provided by NCBI. The rno-miRNA in the rat miRbase and the predicted miRNA were performed, respectively, to screen differential expression and subsequent analysis.

### 2.11. Prediction of Target Genes

The mature miRNA is composed of longer primary transcripts by a series of nucleases cleaved and then assembled into the RNA-induced silencing complex, by means of complementary base pairing to identify target genes and the degree of complementarity of different guiding silencing complex degradation of target mRNA or inhibit translation of the target mRNA. Based on the trend of mRNA, circRNA, and ncRNA, we used the internationally recognized miRNA target gene prediction algorithm Miranda to predict the negative correlation trend.

### 2.12. Series Cluster

We obtained the union of all differentially expressed RNA (including mRNA, circRNA, ncRNA, and lncRNA) of the DM group vs. NC group, treatment groups vs. NC group, and treatment groups vs. DM groups. Then, according to the measured signal value of sequencing to analyze the state trend on the set of RNA showed that the minimum correlation coefficient is 0.85. The significant state trend expression profiles of the set of RNA were identified and focused on the RNA associated with these trends. Drug-related expression trends is that the gene expression levels between the DM group vs. NC group and the treatment group vs. DM group show the opposite trend, and the expression level in the NC group is consistent with the treatment group.

### 2.13. Analysis of Gene Function

We analyzed the acquired genes by GO and pathway enrichment based on the DAVID database to obtain all GO and pathway of related genes. Fisher test was used to calculate the significant level of each GO and pathway (*P* value). The results of multiple hypothesis test are corrected, and the misjudgment rate (FDR) is obtained. If a test function value *P* <0.05, then the function of this gene is significantly enriched.

### 2.14. Construction of Functional Regulatory Network

GO-Tree is constructed based on the gene ontology directed acyclic graph to provide user friendly data navigation and visualization. We selected the significant GO-Term (*P* value <0.01) in GO Analysis based on the up and down differentially expressed genes to construct the GO-Tree to summarize the function affected in the experiment. Taking the genes under the trend, we focused to do GO-Analysis, and the significant GO-Term (*P* value <0.05) is used as the research object to perform functional regulation analysis and construct a regulatory network. The picture deletes which part of the two terms has a hierarchical subordinate relationship and no relation to other term, but are not deleted in the list of relationships. Pathway analysis was used to find out the significant pathway of the differential genes according to the KEGG database. We turn to Fisher's exact test to select the significant pathway, and the threshold of significance was defined by*P* value and FDR. We picked the genes in the enriched biological pathway and used Cytoscape for graphical representations of pathways.

### 2.15. ceRNA Network

miRNA can cause degradation and hinder the protein translation of mRNA and influence the function of important proteins by influencing the structure of mRNA and protein. Therefore, the changes in the expression of miRNA and mRNA should be negatively correlated. At the same time, miRNA will combine with circRNA and ncRNA, thereby affecting the regulatory role of the same miRNA on mRNA. Extracting miRNA that regulates both mRNA and circRNA simultaneously shows there is a positive correlation between the trend expression of mRNA and circRNA. Other ncRNA was analyzed as above. Select the mRNA contained in the significant entries of the GO pathway analysis based on the results of miRNA, and GO pathway significant genes are intersected. Based on the list of ceRNA relationships of intersection genes, circRNA-miRNA-mRNA and ncRNA-miRNA-mRNA network diagrams are drawn.

### 2.16. Weighted Gene Coexpression Network Analysis

Coexpression means that the expression patterns of two genes have high similarity in a set of samples. The basic mechanism of the coexpression network is to extract the signal value of each gene, calculate the Pearson correlation between two genes, and set threshold. When the Pearson correlation exceeds the threshold between the two genes, there is a coexpression relationship between the two genes. Finally, we construct a network with all coexpression genes, which is a coexpression network. The WGCNA algorithm is a typical system biology algorithm for constructing a gene coexpression network, which is based on high-throughput gene mRNA expression data and is widely used in the field of international biomedicine. Taking trend mRNA and ncRNA and trend mRNA and circRNA as the research object, the weight coexpression analysis was carried out using the WGCNA algorithm.

### 2.17. Statistical Analysis of Biological Experimental Results

Biological experimental results were performed using IBM SPSS statistics 20.0. ALL biological experimental results were presented as mean ± standard deviation (SD). Comparisons among groups were performed using one-way ANOVA. Values of *P* < 0.05 were considered statistically significant.

## 3. Results

### 3.1. Comparison of Body Weight, FBG, and RBG in SP, FJG, and SP-FJG Groups

As shown in Table 1, compared with the NC group, the body weight of all ZDF (fa/fa) rats was increased significantly since the first week (*P* < 0.01); compared with the DM group, body weight of the SP group significantly decreased at the fourth week (*P* < 0.05). The SP-FJG group body weight decreased significantly from the beginning of the fourth week (*P* < 0.05). It shows that the SP group and the SP-FJG group have the effect of reducing body weight.

Compared with the NC group, the FBG and RBG of the DM group increased significantly from the first week (*P* < 0.01). In comparison with the DM group, FBG decreased significantly from the beginning of the third week in the SP group (all *P* < 0.01) and RBG decreased significantly at the third, fifth, and sixth week (*P* < 0.05). As for the FJG group, FBG decreased at the beginning of the fourth week (all *P* < 0.01), while RBG decreased at the fourth, fifth, and sixth week (*P* < 0.05). In the SP-FJG group, FBG decreased significantly at the fourth, fifth, and sixth week (*P* < 0.05) and RBG decreased significantly from the beginning of the second week to the sixth week (*P* < 0.05). In addition, the sitagliptin phosphate (SP group) and combined medication (SP-FJG group) had significant effect of reducing FBG, and the effect of combined medication on reducing RBG was better than monotherapy (Table 1).

### 3.2. FJG Combined with SP Improved Glucose Tolerance and Insulin Tolerance Better than Individual Treatment

In the OGTT, the blood glucose values of DM rats compared with the NC group were significantly increased (*P* < 0.01), and the AUC was significantly increased (*P* < 0.01) as well. Compared with the DM group, the blood glucose values of all three treatment groups decreased at 0 min and 60 min (*P* < 0.05), and the value of blood glucose of 30 min and 120 min in the SP-FJG group was significantly decreased (*P* < 0.05). The AUC of all three treatment groups was decreased significantly (*P* < 0.05), while AUC of the SP-FJG group was significantly downregulated compared with the SP group. These data indicated that the effects of combination of drugs on improving glucose tolerance were better than that of the individual drugs (Table 2).

We performed ITT, and the blood glucose values and AUC of the DM group compared with the NC group were similar with OGTT experiments. Compared with the DM group, the blood glucose of SP, FJG, and SP-FJG groups was significantly decreased (*P* < 0.05) as well as AUC (*P* < 0.01). In the SP-FJG group, the blood glucose values of 30 min, 60 min, and 120 min and AUC were the lowest of all the three treatment, indicating that both SP as the monotherapy group (SP group) and the adjunctive therapy group (SP-FJG group) have the effect of increasing insulin sensitivity, but the combination of drugs was better (Table 2).

### 3.3. FJG Combined with SP Decreased FINS and Improved Insulin Sensitivity

Compared with the NC group, FINS and HOMA-IR in the DM group were notably increased at the sixth week (*P* < 0.01). Compared with DM, there was no significant change in FINS of SP and FJG groups (*P* > 0.05), while FINS in the SP-FJG group was dramatically lowered (*P* < 0.01). In addition, compared to DM, the HOMA-IR of the SP group decreased greatly (*P* < 0.05) and for FJG and SP-FJG groups (*P* < 0.01); the SP-FJG group was lower than the SP group but not significant (Table 3).

### 3.4. Comparison of Serum Detection in SP, FJG, and SP-FJG Groups

Compared with the NC group, TNF-α and IL-6 of the DM group were significantly increased (*P* < 0.01). Compared with the DM group, only in the SP-FJG group TNF- α and IL-6 were significantly decreased (*P* < 0.05), indicating that SP-FJG combination therapy reduced the inflammatory state in rats better than that of monotherapy (Table 4).

Compared with the NC group, HDL was significantly decreased (*P* < 0.01) while LDL, TC, and TG raised significantly (*P* < 0.01) in the DM group; compared with the DM group, HDL, LDL, and TC of the SP group increased (*P* < 0.05) and TG decreased (*P* < 0.01). In the FJG group, TC and TG were decreased (*P* < 0.05), and LDL distinctly decreased. In the SP-FJG group, TG was significantly lowered (*P* < 0.01). Notably, the increased LDL and TC in the SP group were attenuated with the combination group, indicating that combination therapy had better effect in lipid metabolism than monotherapy (Table 4).

Compared with the NC group, SOD of the DM group significantly decreased while MDA increased significantly (*P* < 0.01). Compared to the DM group, SOD in the SP group, the FJG group, and the SP-FJG group increased significantly while MDA decreased significantly (*P* < 0.01). It shows that combination therapy and individual treatments can improve the state of oxidative stress (Table 4).

### 3.5. Differential Expression Analysis in SP, FJG, and SP-FJG Groups

According to the expression of RNA, the differentially expressed RNAs were screened by the algorithm EB and conformed to the standard of |log 2(Fold Change)| > 0.667 and the FDR < 0.05. If log^2^ FC > 0.667, the RNA is up while log^2^ FC < −0.667 is down. In different cases, the number of differentially expressed RNAs is shown in Table 5. Correspondingly, 134, 1026, and 621 differentially expressed genes (DEGs) were detected in the liver for SP vs. DM, FJG vs. DM, and SP-FJG vs. DM, details are given in Table 5.

### 3.6. Expression Trend Analysis in SP, FJG, and SP-FJG Groups

**Figure 1:**
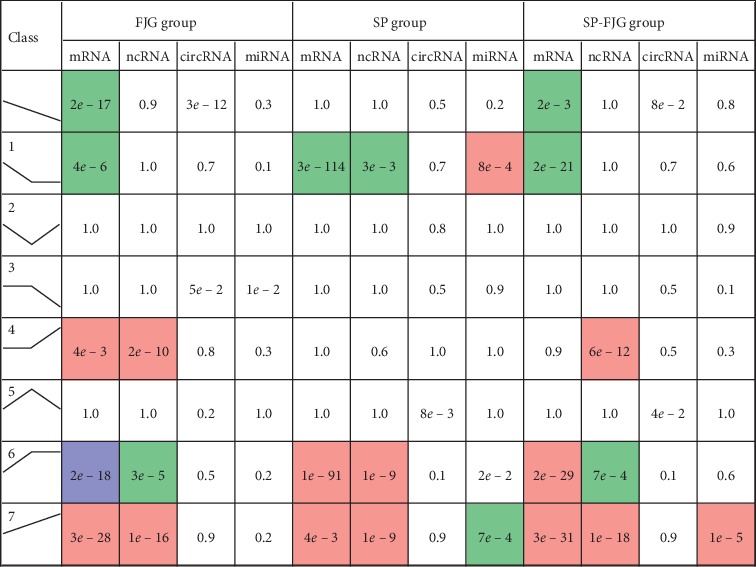
Trend analysis of differential expression of RNA.

**Figure 2:**
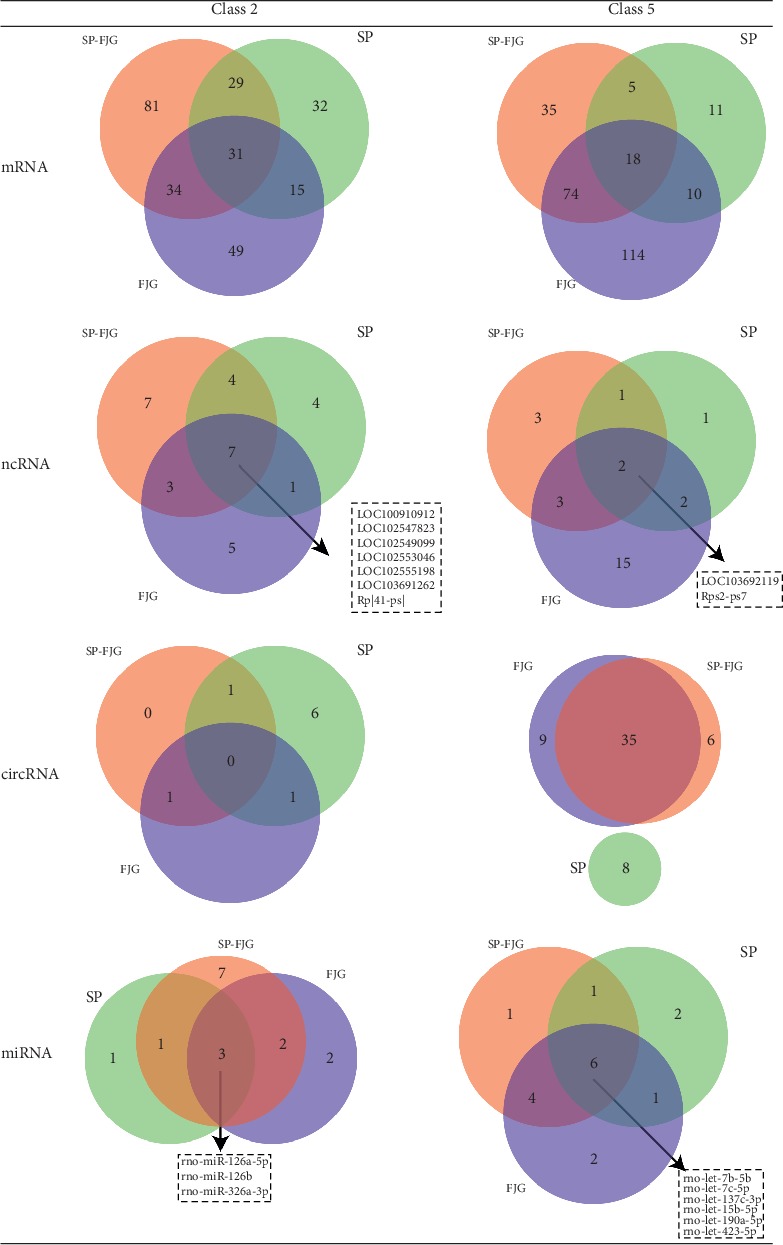
The relationship between the pharmacodynamic trends of RNA in different treatment groups. The left is the four RNA; the corresponding RNA is in the Wayne map of the three treatments, and the box in the Wayne map indicates the RNA expressed in all of the three treatments.

We conducted a trend analysis of the differential expression of RNA in the SP group, the FJG group, and the SP-FJG group according to the RNA category (Figure 1). In view of the trend of expression, trend 2 and 5 are related to the effects of drugs. And then, we made a statistical analysis of the two trends of RNA, as shown in Figure 2.

### 3.7. Gene Function Analysis of the Relationship between Pharmacodynamics Trend of RNA in SP, FJG, and SP-FJG Groups

**Figure 3:**
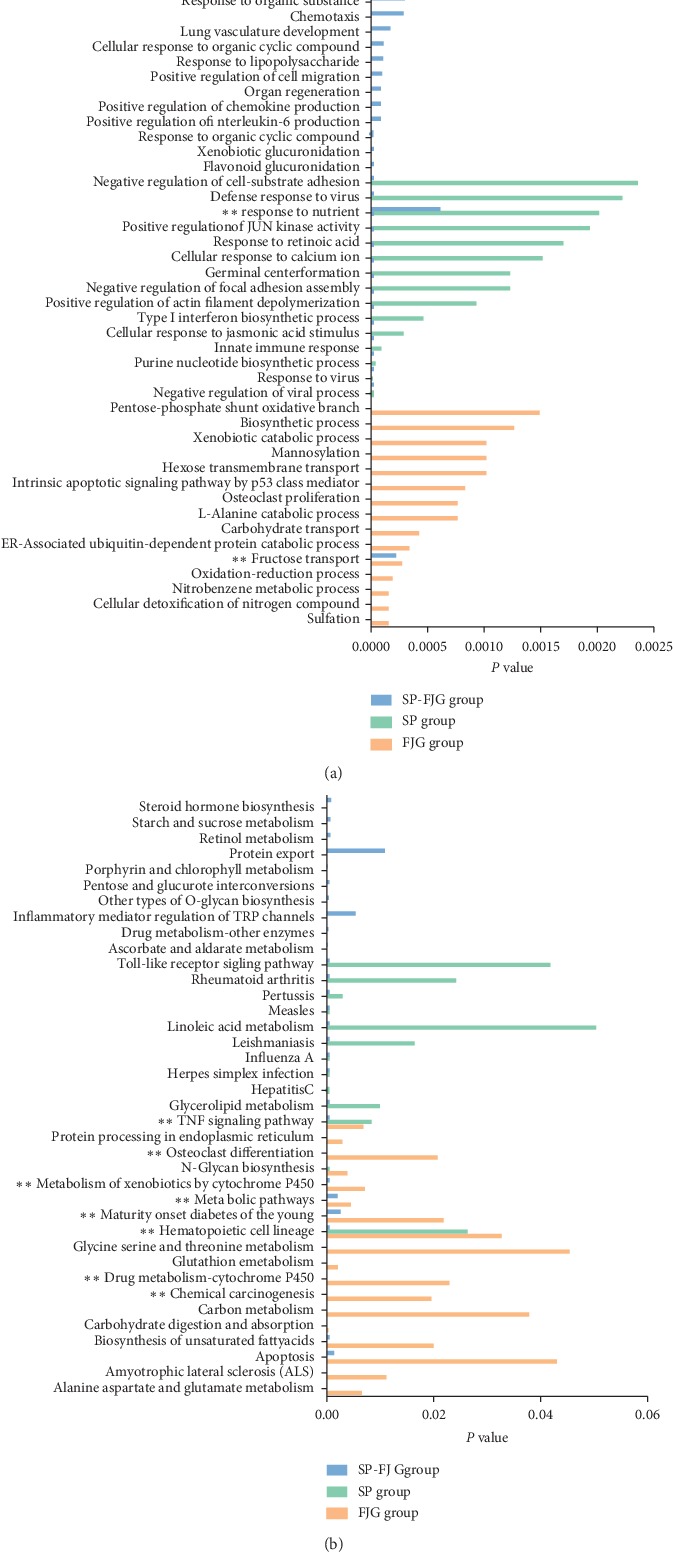
Significant enrichment of GO BP and pathway in different treatment groups. (a) Significantly enriched GO BP; (b) significantly enriched pathway.

Biological function of drug effect trend gene in different therapeutic effects was analyzed. Functional enrichment analysis of the genes in trend 2 and 5 was performed. The significant enrichment gene ontology terms in biological process (GO BP) and pathways in different treatments are shown in Figures 3(a) and 3(b), respectively. The trend genes of FJG are mainly associated with sulfation, cellular detoxification of nitrogen compound, etc. The efficacy genes of the SP group mainly focus on the biological process, for example, purine nucleotide biosynthetic process, innate immune response, and cellular response to jasmonic acid stimulus. The trend genes of the SP-FJG group were mainly involved in biological processes as flavonoid glucuronidation, xenobiotic glucuronidation, and cellular response to organic cyclic compound. Effect genes of both SP group and SP-FJG group regulate the process in response to nutrient. The biological processes of the fructose transport is regulated by both the SP-FJG drugs and FJG. According to the biological pathway of pharmacophore regulation, the pathway of TNF signaling, and hematopoietic cell lineage, the pharmacophores are regulated by both FJG and SP. The trend genes of the SP-FJG group and the FJG group both regulated the metabolism of xenobiotics by cytochrome P450, metabolic pathways, maturity onset diabetes of the young, drug metabolism cytochrome P450, and chemical carcinogenesis pathway. The pharmacophore of SP can specially regulate the toll-like receptor signaling pathway and other pathways. Pharmacophore of FJG specifically regulates alanine aspartate and glutamate metabolism. Apoptosis pathway, ascorbate and aldarate metabolism, and other pathways are regulated by the pharmacophore of the SP-FJG (Figure 3(b)).

**Figure 4:**
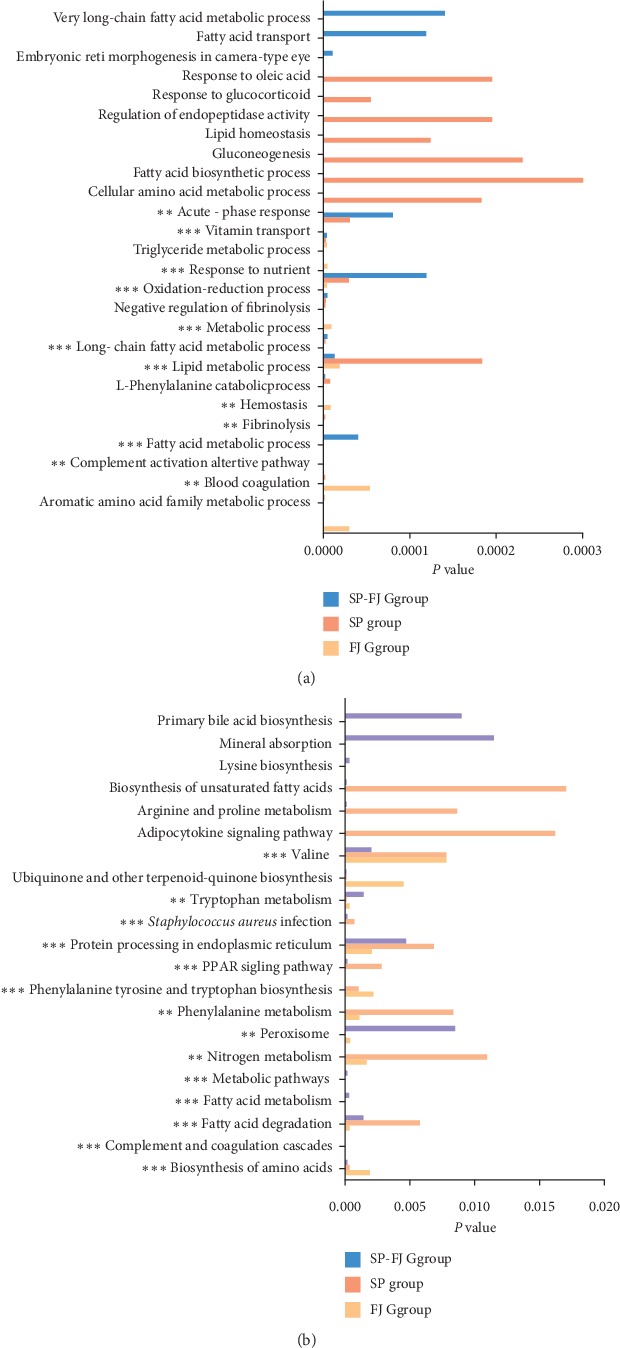
Significant enrichment of GO BP and pathway of circRNA in different treatment groups. (a) Significantly enriched GO BP; (b) significantly enriched pathway.

Then, the GO BP and pathway functions of circRNA were enriched. The biological process enrichment (Figure 4(a)) analysis shows that circRNA efficacy trends of three administration are focused on vitamin transport response to nutrient control, oxidation-reduction, process, and long-chain fatty acid metabolic process (Figure 4(a)). From the enriched pathway (Figure 4(b)), the treatment groups are mainly involved in metabolic pathways, pathway valine, *Staphylococcus aureus* infection, protein processing in endoplasmic reticulum, PPAR signaling pathway, etc.

### 3.8. Construction of Functional Regulatory Network in SP, FJG, and SP-FJG Groups

**Figure 5:**
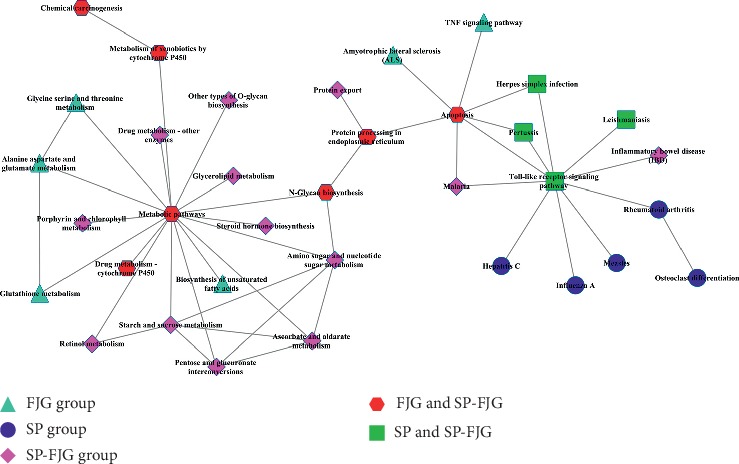
Functional regulatory network of the pathway.

To study the function of regulatory networks of genes involved in different treatment, we constructed a pathway function control network (Figure 5).There are many common pathways in the FJG group and the SP-FJG group, which contain metabolic pathways, drug metabolism cytochrome P450, N-glycan biosynthesis, protein processing in the endoplasmic reticulum, and apoptosis. The apoptosis pathway affects pathway pertussis, herpes simplex infection, and toll-like receptor signaling of SP (Figure 5).

### 3.9. Construction of ceRNA Network in SP, FJG, and SP-FJG Groups

**Figure 6:**
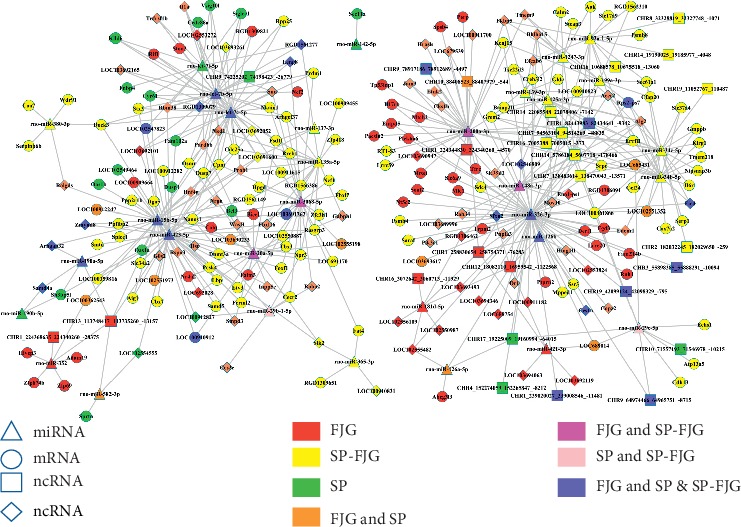
ceRNAs network of different treatments.

The miRNAs of different administration trends were obtained, and then the trends of miRNA negatively regulated ncRNA, circRNA, and mRNA were predicted. ceRNA networks of three different treatments were constructed. Then, the topological properties of the network were analyzed and the RNAs of top 5 were obtained. As seen from Table 6, *rno-miR-326-3p, rno-miR-423-5p, rno-miR-15b-5p, rno-let-7c-5p,* and *rno-let-7b-5p* are the top 5 miRNAs. These miRNAs are involved in the three treatments, and rno-miR-326-3p acts on multiple RNAs (Figure 6).

### 3.10. Weighted Coexpression Network Relationship of ncRNA and mRNA

**Figure 7:**
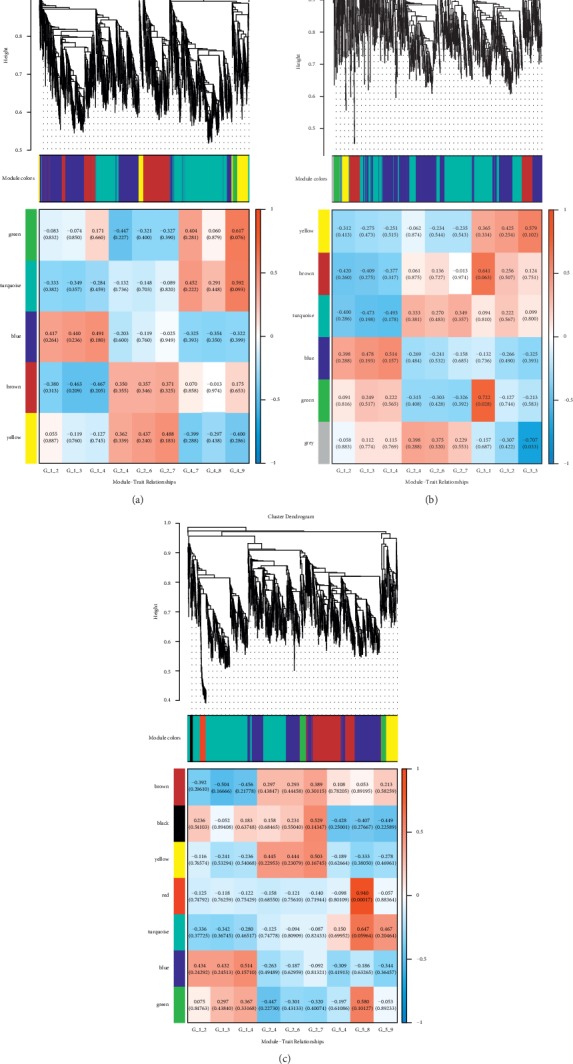
ncRNA and mRNA coexpression analysis of different treatments. (a) WGCNA analysis of the FJG group; (b) WGCNA analysis of the SP group; (c) WGCNA analysis of the SP-FJG group.

**Figure 8:**
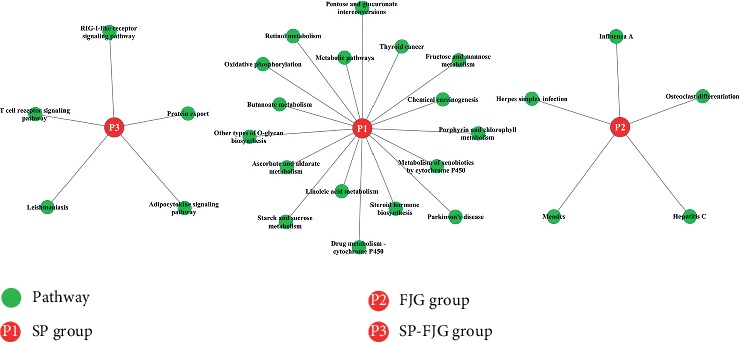
Pathway enrichment analysis of the significant modules. P1 represents SP, P2 represents FJG, and P3 is a combination (SP-FJG). The significant pathway is a green circle, and the significant pathway is *P* < 0.05.

Significant modules were screened to perform the coexpression analysis of ncRNA and mRNA in different treatments. Functional modules are obtained from the above significant modules (Figure 7). The results showed that the SP-FJG group was significantly correlated with red modules, the SP group was significant in the green modules, and the FJG group was also significantly in the green module, and then the three significant modules were analyzed by pathway enrichment (Figure 8).

**Figure 9:**
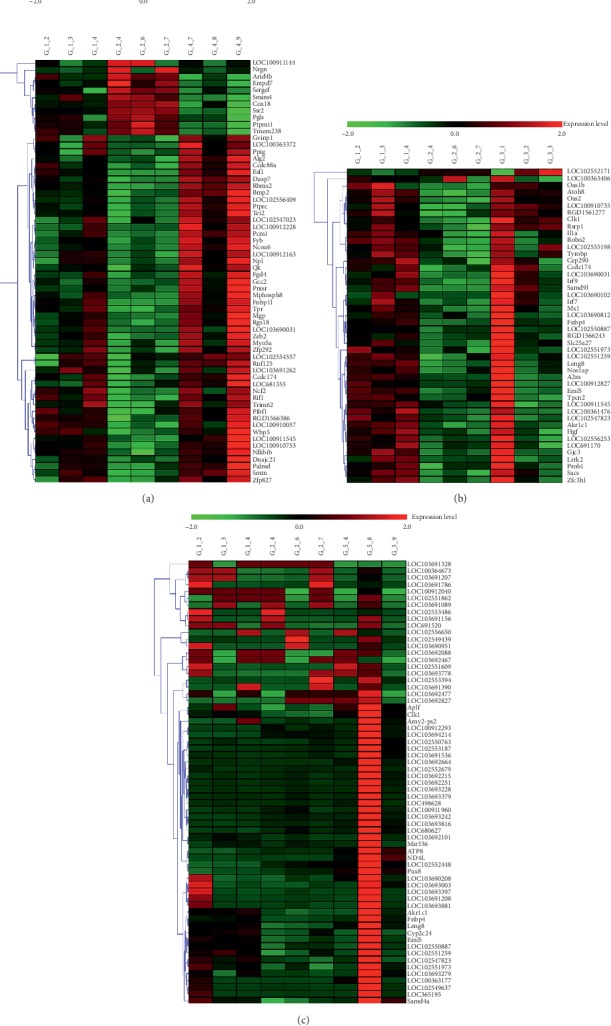
The heatmap of the significant module in different administration. (a) FJG group, (b) SP group, and (c) SP-FGJ group.

Pathway analysis showed that the main regulatory pathways in the SP group were chemical carcinogenesis, metabolism of xenobiotics by cytochrome P450, etc. In the FJG group, that were herpes simplex infection, measles, and so on. The SP-FJG group mainly affected the adipocytokine signaling pathway, T-cell receptor signaling pathway, and RIG-I-like receptor signaling pathway. In addition, the significantly expressed RNA value of three kinds of processing module was extracted, and then a heatmap was generated (Figure 9).

### 3.11. Weighted Correlation Network Analysis of circRNA

**Figure 10:**
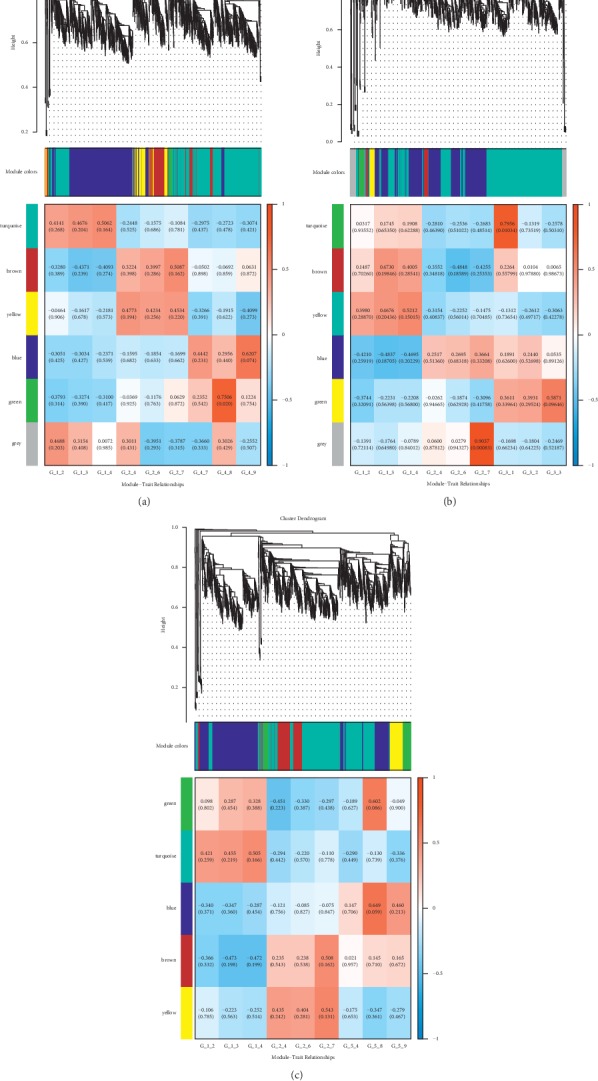
Coexpression of circRNA and mRNA in different administration. (a) WGCNA analysis of the FJG group; (b) WGCNA analysis of the SP group; (c) WGCNA analysis of the SP-FJG group.

**Figure 11:**
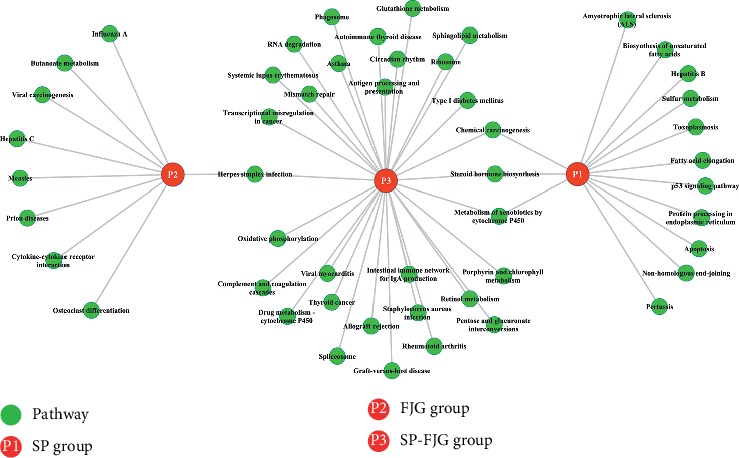
Pathway analysis of significant modules. The SP group is expressed in P1, P2 is the FJG group, and P3 is the SP-FJG group. The significant path is the green round.

Significant modules were screened to perform the coexpression analysis of circRNA and mRNA in different treatments. Functional modules are obtained from the above significant modules (Figure 10). The results showed that the SP-FJG group was significantly correlated with red modules, the SP group and the FJG group were significant in the green modules, and then the three significant modules were analyzed by pathway enrichment to select significantly enriched pathways (*P* value <0.05) (Figure 11).

FJG module mainly regulates butanoate metabolism, osteoclast differentiation, cytokine-cytokine receptor interaction, and other pathways; the SP module mainly controls amyotrophic lateral sclerosis (ALS), p53 signaling pathway, apoptosis, and biosynthesis of unsaturated fatty acids, etc. SP-FJG chiefly controls autoimmune thyroid diseases, type 1 diabetes mellitus, etc. FJG and SP-FJG coregulation pathway is the herpes simplex infection. SP-FJG and SP coregulated pathways including chemical carcinogenesis, steroid hormone biosynthesis, and metabolism of xenobiotics by cytochrome P450.

**Figure 12:**
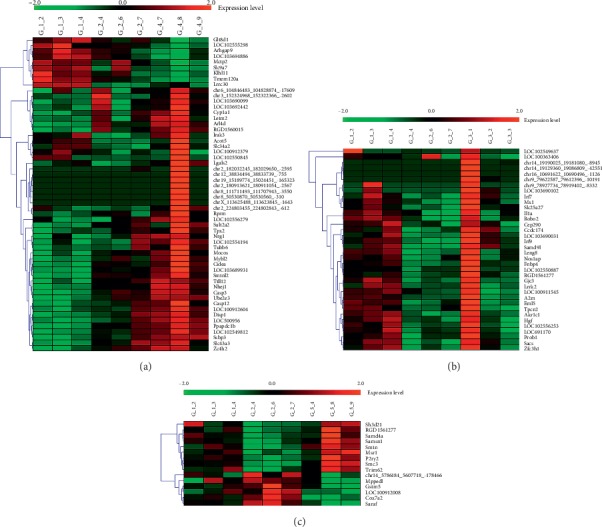
The heatmap of significant module in different administration. (a) FJG group, (b) SP group, and (c) SP-FGJ group.

The significantly enriched pathways were obtained. The expression value of RNA of the green module of the SP group and FJG group and the red module of the SP-FJG group were extracted, and then a heatmap was generated (Figure 12).

### 3.12. ceRNA Network Analysis of Significant Modules

**Figure 13:**
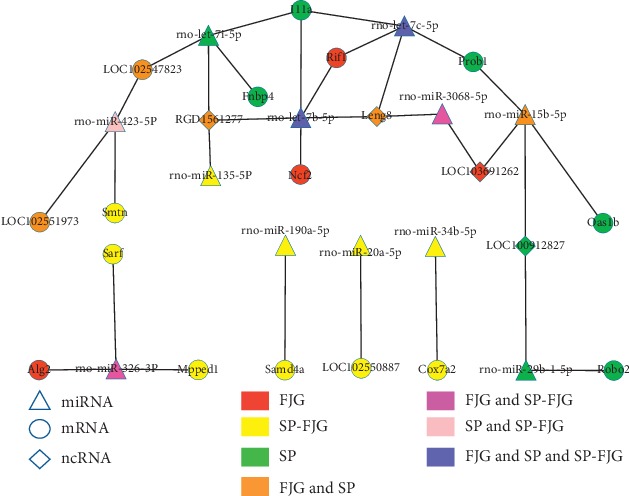
ceRNA network of the significant module in different administration.

To detect clusters of highly interconnected genes and explore the function of ceRNAs on the basis of protein-coding genes, we performed weighted correlation network analysis (WGCNA). From the analysis, we obtained the significant modules of ncRNA and mRNA and circRNA and mRNA. In ncRNA and mRNA, the significantly enriched pathway of the green module of the FJG group, the green module of the SP group, and the red module of the SP-FJG group were obtained. While in circRNA and mRNA, the obviously enriched pathway of the green module of the FJG group, the green module of the SP group, and the blue module of the SP-FJG group were obtained. The ncRNA, mRNA, and circRNA of class 2 and class 5 in the significant modules were extracted, and then the ceRNA networks were constructed (Figure 13).

The ceRNA network analysis showed the top 5 miRNAs in the global ceRNA network which played an important role in the ceRNA of the significant modules. The miRNA *Il1a, Rif1*, and *Leng8* [18] are regulated by *rno-let-7c-5p* as well as *rno-let-7b-5p*, and *Il1a* is a pharmacophore in the SP group and *Rif1* is the effective gene of FJG treatment. *Leng8* is an ncRNA, which have pharmacodynamic function in FJG and SP treatment. The miRNA *rno-let-7c-5p* and *rno-let-7b-5p* had efficacy trends in the three treatments.

### 3.13. Building PPI Network of SP, FJG, and SP-FJG Groups

**Figure 14:**
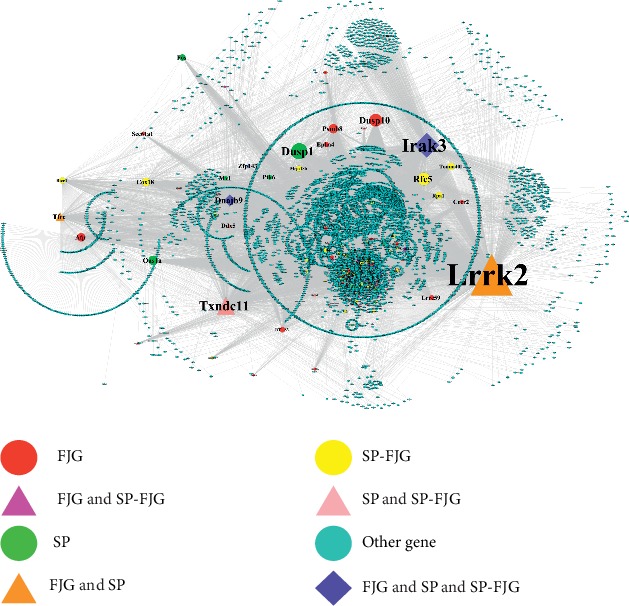
PPI network of the three different administration.

In order to study the role of pharmacophore obtained from different treatments in the biological networks, we obtain the rat protein interaction data from the string database. The data of drug effect trend genes in the three administration groups were obtained, and then the PPI network was constructed (Figure 14).

By analyzing the topological properties of the network, the nodes were arranged in the descending order of degree in topological properties, showing the gene of top 20 (Table 7). *Lrrk2*, a pharmacodynamic gene under the treatment of traditional FJG and SP, got the highest degree in the PPI network and played the most important role in the network. *Irak3* was differentially expressed in the three different treatment groups and was related to drug efficacy, which had a great influence on the network. It can be concluded that *Irak3* is a potential target in drug treatment of disease.

## 4. Discussion

This study is a report of the mechanisms of SP plus FJG in the treatment of diabetes mellitus. Transcriptome and miRNA sequencing were performed to comprehensively elucidate the mechanisms at the genetic level.

In 2007, Aschner et al reported that SP, the first dipeptidyl peptidase 4 (DPP-4) inhibitor, provided a new treatment for patients with type 2 diabetes [19]. SP was proved to be a safe antibiotic medicine that could reduce glycosylated hemoglobin significantly (*P* < 0.001 vs. placebo). FBG, body weight, and systolic blood pressure values were also significantly reduced at week 26 and 52 in the sitagliptin 100 mg group patients compared with placebo (*P* < 0.001). [19, 20]. From the in vivo observation of oral glucose tolerance test by SP, blood glucose level decreased (22.22%) significantly [22].

In recent years, TCMs have been extensively studied in the treatment of T2DM. Xu's et al. study found that compared with the control group, the blood glucose of administration the MDG-1 (300 mg/kg) group decreased by 30% [23]. One study found that administration with high-dose Tangnaikang (TNK) (3.24 g/kg) in SHR/cp rats for 3 weeks, the body weight, and fat mass of SHR/cp rats significantly reduced without affecting food consumption. FBG and FINS in the TNK-treated groups decreased after 6 weeks of treatment. Furthermore, TNK-treated rats exhibited obvious improvements in glucose intolerance and insulin resistance [11]. Another research of *Jiangtang Xiaozhi*, which comprised six commonly used herbs, found that 16 weeks of *Jiangtang Xiaozhi* treatment did not lower fasting blood glucose, but it improved FINS and HDL cholesterol in a Western population with prediabetes or controlled diabetes [24].

In our study, after treatment for 6 weeks, the improvement of FBG, RBG, OGTT, ITT, HOMA-IR, TC, SOD, and MDA were presented by FJG and SP. Combining SP and FJG, not only OGTT, FINS, TNF-*α*, and IL-6 were better improved compared with monotherapy but increased LDL and TC by SP were attenuated when using FJG in combination. Besides, only the combined medication group decreased FINS, TNF-*α*, and IL-6.

In addition, we performed miRNA and transcriptome sequencing of the liver tissue of ZDF rats with different drug treatment. Compared with the DM group, 134, 1026, and 621 DEGs were found in the SP/FJG/SP-FJG group, respectively. According to the type of RNA count, 1248 mRNA, 211 ncRNA, 202 cirRNA, and 120 miRNA were differentially expressed in different groups. In subsequent series cluster analysis, we identified profile 2 and 5 having important biological significance, and these two gene expression trends were related to the drug treatment process. KEGG analysis of the weighted coexpression networks of ncRNA and mRNA and ceRNA indicated that 22 pathways were significantly enriched. PPAR pathway [11], fatty acid metabolism [25], and nitrogen metabolism [26] have been reported to be related to T2DM. Usually, a certain miRNA may have multiple different mRNA targets, whereas a given target gene may also be targeted by multiple miRNAs [27]. In this thesis, by the analysis of the ceRNA network, we found that *rno-miR-326-3p* [28], *rno-miR-423-5p* [29], *rno-miR-15b-5p*, *rno-let-7c-5p*, and *rno-let-7b-5p* were related to pharmacodynamics in different groups.

Wang Y's study confirmed that miR-326-3p targeted on FcγRIII and inhibited its expression under the condition of high glucose, which were associated with glomerular sclerosis of diabetic kidney disease [28]. Another study found that under the condition of obesity, activation of the hepatic NFE2/miR-423-5p axis plays important roles in the progression of type 2 diabetes and NAFLD by repressing the FAM3A-ATP-Akt signaling pathway [29]. Besides, a study found the elevated serum miR-423-5p combined with oxidized lipoprotein can be used as novel biomarkers for potential auxiliary diagnosis of T2DM patients and T2DM patients with microvascular complications [30]. *MiR-15b* maybe a potential therapeutic target for therapeutics for the diabetic retina. It plays a major role in the inhibition of insulin resistance via reduced *TNFα* and *SOCS3* signaling and increased IGFBP-3 levels, resulting in REC protection from hyperglycemia-induced apoptosis [31].

By using the PPI and coexpression networks of the transcriptomes in different treatment groups, we generated highly connected modules which were used to enrich the gene mutations in ZDG rats using the WGCNA algorithm. Using the string database, PPI network analysis found *Lrrk2, Irak3,* and other 20 genes, and these genes may be pharmacodynamic genes. The 3′UTR of the *Lrrk2* gene would have been a target of *miR-712* and then dampens the phosphorylation of p38 and ERK1/2 kinases. However, *miR-712* restored insulin-stimulated glucose uptake by myoblasts through downregulating macrophage-mediated inflammatory response [32]. A study in 123 patients and 46 age-matched controls reported the addition of reactive oxygen species, obesity, low adiponectin, and high glucose and interleukin-6 as cause of the abatement in *Irak3* in THP-1 cells in vitro [33]. *Irak3* is a crucial inhibitor of inflammation, obesity, and metabolic syndrome. And chronic low-grade inflammation is now considered to have a vital role in the development of obesity and related metabolic diseases such as T2DM, insulin resistance, and the metabolic syndrome and cardiovascular disease [34, 35].

## 5. Conclusion

In conclusion, the effect of SP-FJG in regulating glucose tolerance, TNF-*α*, IL-6, FINS, and hyperlipidemia was better than using SP individually in ZDF rats. We conducted a comprehensive comparison of gene expression treated with SP, FJG, and SP-FJG. We identified that many genes may be responsible for T2DM by transcriptome and miRNA sequencing. In combination with the sequencing results, we speculated that 1248 mRNAs, 211 ncRNA, 202 cirRNA, and 120 miRNA were related to the treatment of SP and FJG for diabetic. By ceRNA, *rno-miR-326-3p*, *rno-miR-423-5p*, *rno-miR-15b-5p*, *rno-let-7c-5p*, and *rno-let-7b-5p* were connected with pharmacodynamics in different groups. PPI network analysis discovered that *Lrrk2, Irak3,* and other 18 genes may be pharmacodynamic genes. This study provides the basis for functional study of diabetes-related genes and the molecular mechanism of T2DM.

## Figures and Tables

**Table 1:** Measure of body weight, FBG, and RBG of rats in NC, DM, SP, FJG, and SP-FJG groups.

	Group	1st week	2nd week	3rd week	4th week	5th week	6th week
Body weight (g)	NC	284.3 ± 11.82^*∗∗*^	309.3 ± 44.57^*∗∗*^	297.6 ± 11.78^*∗∗*^	310.1 ± 10.76^*∗∗*^	322.8 ± 9.93^*∗∗*^	316.2 ± 9.66^*∗∗*^
	DM	331.2 ± 18.09	352.4 ± 13.07	363.15 ± 23.32	375.13 ± 29.89	374.4 ± 29.08	378.6 ± 52.48
	SP	320.8 ± 16.6	342.37 ± 22.5	346.46 ± 14.33	344.4 ± 17.95^*∗*^	357.7 ± 21.14	343.94 ± 25.17
	FJG	331.3 ± 18.05	337.8 ± 17.77	345.8 ± 19.28	353.1 ± 26.55	356.6 ± 22.71	342.8 ± 26.75
	SP-FJG	341.6 ± 21.60	353.1 ± 16.86	354.9 ± 22.03	346.64 ± 21.3^*∗*^	344.5 ± 16.73^*∗*^	324.09 ± 18.95^*∗∗*^

FBG (mmol/L)	NC	3.71 ± 0.39^*∗∗*^	3.59 ± 0.21^*∗∗*^	3.43 ± 0.18^*∗∗*^	3.41 ± 0.17^*∗∗*^	4.03 ± 0.29^*∗∗*^	3.7 ± 0.39^*∗∗*^
	DM	4.68 ± 0.93	5.6 ± 0.99	7.79 ± 1.79	8.38 ± 2.12	11.53 ± 2.63	12.65 ± 3.31
	SP	4.64 ± 0.86	4.96 ± 0.86	5.68 ± 1.26^*∗∗*^	5.36 ± 0.92^*∗∗*^	5.43 ± 1^*∗∗*^	7.31 ± 2.49^*∗∗*^
	FJG	4.55 ± 0.69	5.11 ± 0.63	6.45 ± 1.73	5.47 ± 1.29^*∗∗*^	6.12 ± 1.07^*∗∗*^	6.9 ± 0.77^*∗∗*^
	SP-FJG	4.7 ± 0.51	4.8 ± 0.72	6.69 ± 2.45	5.32 ± 1.19^*∗∗*^	5.45 ± 1.27^*∗∗*^	7.44 ± 1.12^*∗∗*^

RBG (mmol/L)	NC	5.04 ± 0.26^*∗∗*^	4.21 ± 0.23^*∗∗*^	4.15 ± 0.24^*∗∗*^	3.66 ± 0.14^*∗∗*^	3.48 ± 0.27v	3.61 ± 0.12^*∗∗*^
	DM	18.58 ± 3.24	20.07 ± 3.71	19.88 ± 2.8	22.25 ± 2.85	23.03 ± 4.85	24.84 ± 4.29
	SP	17.6 ± 4.75	17.08 ± 3.8	18.75 ± 3.36^*∗*^	19.45 ± 3.54	17.06 ± 2.04^*∗∗*^	18.4 ± 2.31^*∗∗*^
	FJG	19.08 ± 4.55	16.87 ± 3.2	18.2 ± 3.56	18.07 ± 4.3^*∗*^	17.08 ± 2.96^*∗∗*^	19.27 ± 2.25^*∗∗*^
	SP-FJG	18.44 ± 4.24	16.34 ± 3.76^*∗*^	15.85 ± 4.17^*∗*^	16.92 ± 2.1^*∗∗*^	14.92 ± 4.9^*∗∗*^	16.65 ± 3.18^*∗∗*^

All values represent the means ± SD (*n* = 10). ^*∗*^Significant difference with the DM group designated as *P* < 0.05. ^*∗∗*^Significant difference with the DM group designated as *P* < 0.01. ^#^Significant difference with the SP group designated as *P* < 0.05. ^##^Significant difference with the SP group designated as *P* < 0.01.

**Table 2:** Measure of OGTT and ITT of rats in NC, DM, SP, FJG, and SP-FJG groups.

	Group	Blood glucose (mmol/L)				AUC
		0 min	30 min	60 min	120 min	
OGTT	NC	3.72 ± 0.47^*∗∗*^	6.24 ± 2^*∗∗*^	4.46 ± 0.48^*∗∗*^	3.42 ± 0.37^*∗∗*^	9.11 ± 1.04^*∗∗*^
	DM	11.24 ± 2.06	23.48 ± 3.37	27.04 ± 3.95	22.64 ± 5.73	46.15 ± 5.74
	SP	8.4 ± 1.08^*∗*^	20.08 ± 1.63	21.56 ± 2.17^*∗*^	17.68 ± 2.02	37.15 ± 1.54^*∗*^
	FJG	7.84 ± 1.39^*∗*^	21.1 ± 2.83	20.52 ± 2.15^*∗*^	17.94 ± 4.29	36.87 ± 2.77^*∗*^
	SP-FJG	7.94 ± 1.43^*∗*^	17.98 ± 2.17^*∗*^	19.04 ± 3.1^*∗*^	14.62 ± 1.67^*∗*#^	32.57 ± 3.15^*∗∗*#^

ITT	NC	3.02 ± 0.17^*∗∗*^	1.88 ± 0.44^*∗∗*^	1.66 ± 0.41	2.04 ± 0.6	3.96 ± 0.45
	DM	16.7 ± 2.77	12.34 ± 1.69	8.64 ± 1.21	10.46 ± 1.81	22.06 ± 2.17
	SP	7.8 ± 0.66^*∗∗*^	4.68 ± 0.61^*∗∗*^	4.36 ± 0.85^*∗∗*^	5.3 ± 0.87^*∗∗*^	10.21 ± 1.02^*∗∗*^
	FJG	10.1 ± 1.34^*∗∗*^	7.08 ± 0.83^*∗∗*^	6.26 ± 1.8^*∗*^	6.08 ± 0.63^*∗∗*^	13.8 ± 1.56^*∗∗*^
	SP-FJG	9.76 ± 2.25^*∗∗*^	3.88 ± 0.42^*∗∗*^	4 ± 0.71^*∗∗*^	4.38 ± 0.64^*∗∗*^	9.57 ± 1.08^*∗∗*^

All values represent the means ± SD (*n* = 5). ^*∗*^Significant difference with the DM group designated as *P* < 0.05. ^*∗∗*^Significant difference with the DM group designated as *P* < 0.01. ^#^Significant difference with the SP group designated as *P* < 0.05. ^##^Significant difference with the SP group designated as *P* < 0.01.

**Table 3:** FINS and HOMA-IR of rats in NC, DM, SP, FJG, and SP-FJG groups.

Group	FINS (uIU/ml)	HOMA-IR
NC	13.00 ± 0.40^*∗∗*^	2.14 ± 0.25^*∗∗*^
DM	21.48 ± 2.22	12.19 ± 4.47
SP	23.84 ± 5.62	7.98 ± 3.37^*∗*^
FJG	24.21 ± 3.26	7.37 ± 1.21^*∗∗*^
SP-FJG	17.32 ± 1.55^*∗∗*^	5.69 ± 1.30^*∗∗*^

All values represent the means ± SD (*n* = 7). ^*∗*^Significant difference with the DM group designated as *P* < 0.05. ^*∗∗*^Significant difference with the DM group designated as *P* < 0.01. ^#^Significant difference with the SP group designated as *P* < 0.05. ^##^Significant difference with the SP group designated as *P* < 0.01.

**Table 4:** Measure of serum targets in NC, DM, SP, FJG, and SP-FJG groups.

Group	TNF-*α* (pg/ml)	IL-6 (pg/ml)	HDL (mmol/L)	LDL (mmol/L)	TC (mmol/L)	TG (mmol/L)	SOD (U/ml)	MDA (nmol/ml)
NC	56.13 ± 3.82^*∗∗*^	107.88 ± 10.53^*∗∗*^	1.71 ± 0.17^*∗∗*^	0.29 ± 0.08^*∗∗*^	1.99 ± 0.21^*∗∗*^	0.31 ± 0.11^*∗∗*^	80.39 ± 4.25^*∗∗*^	4.50 ± 0.58^*∗∗*^
DM	74.37 ± 3.79	151.73 ± 5.62	1.42 ± 0.24	1.58 ± 0.19	2.92 ± 0.29	2.86 ± 0.11	71.64 ± 2.73	6.91 ± 0.69
SP	75.58 ± 5.30	153.08 ± 9.63	1.94 ± 0.20^*∗∗*^	1.80 ± 0.26^*∗*^	3.20 ± 0.22^*∗*^	2.31 ± 0.47^*∗∗*^	87.90 ± 3.59^*∗∗*^	5.07 ± 0.95^*∗∗*^
FJG	78.89 ± 6.32	159.44 ± 17.62	1.44 ± 0.20	1.40 ± 0.27	2.56 ± 0.37^*∗∗*^	2.46 ± 0.48^*∗*^	100.07 ± 9.60^*∗∗*^	4.72 ± 0.41^*∗∗*^
SP-FJG	69.01 ± 7.18^*∗*#^	133.02 ± 17.92^*∗∗*##^	1.59 ± 0.12	1.44 ± 0.15	2.79 ± 0.13	2.39 ± 0.35^*∗∗*^	89.11 ± 6.69^*∗∗*^	4.82 ± 0.41^*∗∗*^

All values represent the means ± SD (*n* = 7). ^*∗*^Significant difference with the DM group designated as *P* < 0.05. ^*∗∗*^Significant difference with the DM group designated as *P* < 0.01. ^#^Significant difference with the SP group designated as *P* < 0.05. ^##^Significant difference with the SP group designated as *P* < 0.01.

**Table 5:** All the DEGs in different comparisons.

	mRNA		ncRNA		cirRNA		miRNA		Total
	UP	Down	Up	Down	Up	Down	Up	Down	
DM vs. NC	513	572	73	45	49	29	29	29	1339
SP vs. DM	41	21	7	2	19	30	1	13	134
SP vs. NC	435	463	80	29	24	29	39	36	1135
FJG vs. DM	516	259	102	23	28	41	27	30	1026
FJG vs. NC	1000	784	194	58	40	46	49	60	2231
SP-FJG vs. DM	269	142	58	19	32	52	23	26	621
SP-FJG vs. NC	732	621	164	34	43	45	50	48	1737

**Table 6:** Topological features for top 5 RNAs in the ceRNA network.

Name	Degree	Average shortest path length	Betweenness centrality	Closeness centrality	Neighborhood connectivity	Radiality	Topological coefficient
rno-miR-326-3p	61	2.0423	0.5409	0.4897	2.4098	0.8263	0.1007
rno-miR-423-5p	37	2.3643	0.3499	0.4230	2.3243	0.7726	0.0883
rno-miR-15b-5p	37	2.4214	0.3353	0.4130	2.4324	0.7631	0.1102
rno-let-7c-5p	35	2.5643	0.1614	0.3900	3.3429	0.7393	0.2130
rno-let-7b-5p	35	2.6214	0.1533	0.3815	3.2000	0.7298	0.2444

**Table 7:** Topological features for top 20 genes in the PPI network.

Name	Degree	Average shortest path length	Betweenness centrality	Closeness centrality	Clustering coefficient	Topological coefficient
*Lrrk2*	2413	1.9294	0.3861	0.5183	0.0022	0.0026
*Irak3*	1387	2.1311	0.1271	0.4692	0.0039	0.0032
*Txndc11*	968	2.2135	0.1126	0.4518	0.0046	0.0038
*Rfc5*	652	2.6483	0.0679	0.3776	0.0022	0.0066
*Dusp1*	848	2.3084	0.0644	0.4332	0.0066	0.0044
*Afp*	393	2.7355	0.0565	0.3656	0.0023	0.0069
*Tfrc*	416	2.6417	0.0563	0.3785	0.0027	0.0066
*Dnajb9*	592	2.3182	0.0549	0.4314	0.0062	0.0044
*Psmb8*	443	2.7090	0.0510	0.3691	0.0024	0.0077
*Oas1a*	452	2.1887	0.0461	0.4569	0.0067	0.0077
*Dusp10*	651	2.3613	0.0320	0.4235	0.0089	0.0051
*Cox18*	398	2.6361	0.0315	0.3793	0.0047	0.0064
*Bicc1*	267	2.8185	0.0308	0.3548	0.0015	0.0113
*Tomm40 l*	321	2.7891	0.0288	0.3585	0.0031	0.0096
*RT1-S3*	217	2.7531	0.0259	0.3632	0.0052	0.0093
*Mrps18b*	231	2.9355	0.0258	0.3407	0.0011	0.0137
*Mtx2*	195	2.9554	0.0256	0.3384	0.0024	0.0123
*Rpa1*	316	2.7432	0.0254	0.3645	0.0053	0.0081
*Ddo*	215	2.8658	0.0249	0.3489	0.0020	0.0120
*Sult1b1*	136	2.9094	0.0238	0.3437	0.0021	0.0110

## Data Availability

All data generated or analyzed during this study are included in this published article. The datasets supporting the conclusions of this article are available in the NCBI's Sequence Read Archive repository (PRJNA523690: https://www.ncbi.nlm.nih.gov/bioproject/523690).
